# Supplementary material for: Deep neural networks enable quantitative movement analysis using single-camera videos
Source: Nat Commun. 2020 Aug 13;11:4054. doi: 10.1038/s41467-020-17807-z (PMC7426855; doi:10.1038/s41467-020-17807-z)
Supplement: Supplementary file 2 — Reporting Summary [file 41467_2020_17807_MOESM2_ESM.pdf]

nature research

Corresponding author(s): Łukasz Kidziński
Michael Schwartz

Last updated by author(s): Apr 13, 2020

Reporting Summary

Nature Research wishes to improve the reproducibility of the work that we publish. This form provides structure for consistency and transparency in reporting. For further information on Nature Research policies, see Authors & Referees and the Editorial Policy Checklist .

Statistics

For all statistical analyses, confirm that the following items are present in the figure legend, table legend, main text, or Methods section.

n/a	Confirmed	
☐	☒	The exact sample size (*n*) for each experimental group/condition, given as a discrete number and unit of measurement
☐	☒	A statement on whether measurements were taken from distinct samples or whether the same sample was measured repeatedly
☐	☒	The statistical test(s) used AND whether they are one- or two-sided<br>*Only common tests should be described solely by name; describe more complex techniques in the Methods section.*
☐	☒	A description of all covariates tested
☐	☒	A description of any assumptions or corrections, such as tests of normality and adjustment for multiple comparisons
☐	☒	A full description of the statistical parameters including central tendency (e.g. means) or other basic estimates (e.g. regression coefficient) AND variation (e.g. standard deviation) or associated estimates of uncertainty (e.g. confidence intervals)
☐	☒	For null hypothesis testing, the test statistic (e.g. *F*, *t*, *r*) with confidence intervals, effect sizes, degrees of freedom and *P* value noted<br>*Give P values as exact values whenever suitable.*
☒	☐	For Bayesian analysis, information on the choice of priors and Markov chain Monte Carlo settings
☒	☐	For hierarchical and complex designs, identification of the appropriate level for tests and full reporting of outcomes
☐	☒	Estimates of effect sizes (e.g. Cohen's *d*, Pearson's *r*), indicating how they were calculated

Our web collection on statistics for biologists contains articles on many of the points above.

Software and code

Policy information about availability of computer code

Data collection	The VICON Clinical Manager (VCM) was used to process motion capture data from 12-camera VICON MX system (Vicon,Oxford,UK) at 120Hz.<br>Video recordings of patients were processed using OpenPose 1.2
Data analysis	Custom processing pipeline at Gillette Children's Hosiptal was used to calculate walking speed, cadence. For calculating knee flexion at maximum extension and GDI, engineers used custom code dividing gait cycles into strides, computing gait metrics and averaging them over strides.<br>For processing video data, building neural networks and data analysis we used Python 3.6 with following packages installed:<br>- TensorFlow GPU 2.2.0<br>- Keras 2.3.1<br>- Numpy 1.18.2<br>- ScikitLearn 0.22.2.post1<br>- Pandas 1.0.3<br>- h5py 2.10.0<br>For parts of data analysis we also used R 3.4 with default packages.

For manuscripts utilizing custom algorithms or software that are central to the research but not yet described in published literature, software must be made available to editors/reviewers. We strongly encourage code deposition in a community repository (e.g. GitHub). See the Nature Research guidelines for submitting code & software for further information.

Data

Policy information about availability of data

All manuscripts must include a data availability statement. This statement should provide the following information, where applicable:

- Accession codes, unique identifiers, or web links for publicly available datasets
- A list of figures that have associated raw data
- A description of any restrictions on data availability

Video data used in this study were not publicly available due to restrictions on sharing patient health information. These data were processed by Gillette Specialty Healthcare to a deidentified form using OpenPose software as described in the manuscript. The processed deidentified dataset together with clinical variables used in the paper associated with the processed datapoints, were shared by Gillette Specialty Healthcare and are now publicly available at [LINK AND DOI]. The source data underlying Figs 2 and 3 and Supplementary Figs 2 and 3 are provided as a Source Data file.

Field-specific reporting

Please select the one below that is the best fit for your research. If you are not sure, read the appropriate sections before making your selection.

☒ Life sciences ☐ Behavioural & social sciences ☐ Ecological, evolutionary & environmental sciences

For a reference copy of the document with all sections, see nature.com/documents/nr-reporting-summary-flat.pdf

Life sciences study design

All studies must disclose on these points even when the disclosure is negative.

Sample size	We analyzed a dataset of 1,792 videos of 1,026 unique patients diagnosed with cerebral palsy seen for a clinical gait analysis at Gillette Children's Specialty Healthcare between 1994 and 2015.
Data exclusions	We excluded subjects who were not able to walk with or without an asistive device.
Replication	We provide training code for our machine learning models, the dataset used for training, trained weights of models, as well as a demo that can be used for inference on new datasets.
Randomization	For testing our algorithm we uniformly sampled 10% of participants and validated our methods on that test set.
Blinding	Data was collected in the hospital during routine clinical activities, independently of this post-hoc study. All information regarding clinical condition of subjects was collected following best clinical practice.

Reporting for specific materials, systems and methods

We require information from authors about some types of materials, experimental systems and methods used in many studies. Here, indicate whether each material, system or method listed is relevant to your study. If you are not sure if a list item applies to your research, read the appropriate section before selecting a response.

Materials & experimental systems

n/a	Involved in the study	
☒	☐	Antibodies
☒	☐	Eukaryotic cell lines
☒	☐	Palaeontology
☒	☐	Animals and other organisms
☐	☒	Human research participants
☒	☐	Clinical data

Methods

n/a	Involved in the study	
☒	☐	ChIP-seq
☒	☐	Flow cytometry
☒	☐	MRI-based neuroimaging

Human research participants

Policy information about studies involving human research participants

Population characteristics	Average age in the population was 11 years with the standard deviation (sd) 5.9. Average height was 133 cm (sd 22), and mass 34 kg (sd 17). About half (778) of these patients had multiple gait visits, allowing us to assess the ability of our models to detect longitudinal changes in gait.
Recruitment	Our study included all patients admitted Gillette Children's Specialty Healthcare, who were able to walk with or without an assistive device, who agreed to participate in a research study, and for whom video and motion capture data was taken.
Ethics oversight	University of Minnesota Institutional Review Board

Note that full information on the approval of the study protocol must also be provided in the manuscript.
