# Supplementary material for: Deep neural networks enable quantitative movement analysis using single-camera videos
Source: Nat Commun. 2020 Aug 13;11:4054. doi: 10.1038/s41467-020-17807-z (PMC7426855; doi:10.1038/s41467-020-17807-z)
Supplement: Supplementary file 4 — Description of Additional Supplementary Files [file 41467_2020_17807_MOESM4_ESM.pdf]

Title: Supplementary Movie 1:

Description: Overview of motivation, the algorithm, and its implications. In this video, we discuss the clinical need for cheaper and more accessible movement analysis solutions. Next, we present an example video used in the analysis and walk through the steps of the algorithm. Finally, we show several other example videos covering different severity of gait pathology and we discuss potential applications of the algorithm.
